# Primary repair of transposition of the great arteries with an interrupted aortic arch: a case report and literature review

**DOI:** 10.1186/s13019-020-01177-0

**Published:** 2020-06-11

**Authors:** Qiteng Xu, Shuhua Duan, Pengchao Xing, Rui Chen

**Affiliations:** Heart Center, Qingdao Women and Children’s Hospital, Qingdao University, 6 tongfu Road, Qingdao, Shandong China

**Keywords:** Congenital heart disease, Transposition of the great arteries, Interrupted aortic arch, Primary repair

## Abstract

**Abstract:**

Transposition of the great arteries (TGA) and interruption of the aortic arch (IAA) are uncommon congenital heart diseases. The association between TGA and IAA is rare. The aim of this study is to present a case with combined TGA and IAA, who underwent the primary repair and review the literature with similar cases. The one-month-old patient was admitted with tachypnea and cyanosis. Delayed diagnosis was caused due to the absence of prenatal examination. Echocardiography and computed tomography angiography confirmed TGA with anterior-posterior-oriented great arteries, wide patent ductus arteriosus, type B IAA, ventricular septal defect (VSD) and pulmonary arterial hypertension. The patient underwent a single-stage primary surgical repair process leading to VSD closure, reconstruction of the aortic arch and arterial switch operation in October 2019. The patient is doing well at a 3-month follow-up post-surgery. The echocardiogram suggests a normal systolic function of the ventricles and trivial regurgitation for both aortic and pulmonary valves.

**Conclusions:**

The single-stage repair with VSD closure, reconstruction of aortic arch and arterial switch operation might be an applicable approach for most of the patients with combined TGA and IAA. Long term follow-up is required as a high re-intervention rate for recurrent coarctation, supravalvular aortic stenosis, neoaortic valve regurgitation, obstruction of the right heart system and coronary stenosis has been reported.

## Background

Transposition of the great arteries (TGA) and interruption of the aortic arch (IAA) are uncommon heart abnormalities. The association between TGA and IAA is also occasional. Only about 6% of children with IAA have associated TGA [1]. Surgical treatment, primary repair or two-staged repair of these complex heart anomalies have been reported in several papers. Also, it is often associated with high mortality rates [2]. In this study along with a detailed literature review, we have also described a case where the patient underwent primary repair of TGA with IAA.

## Case report

**Fig. 1:**
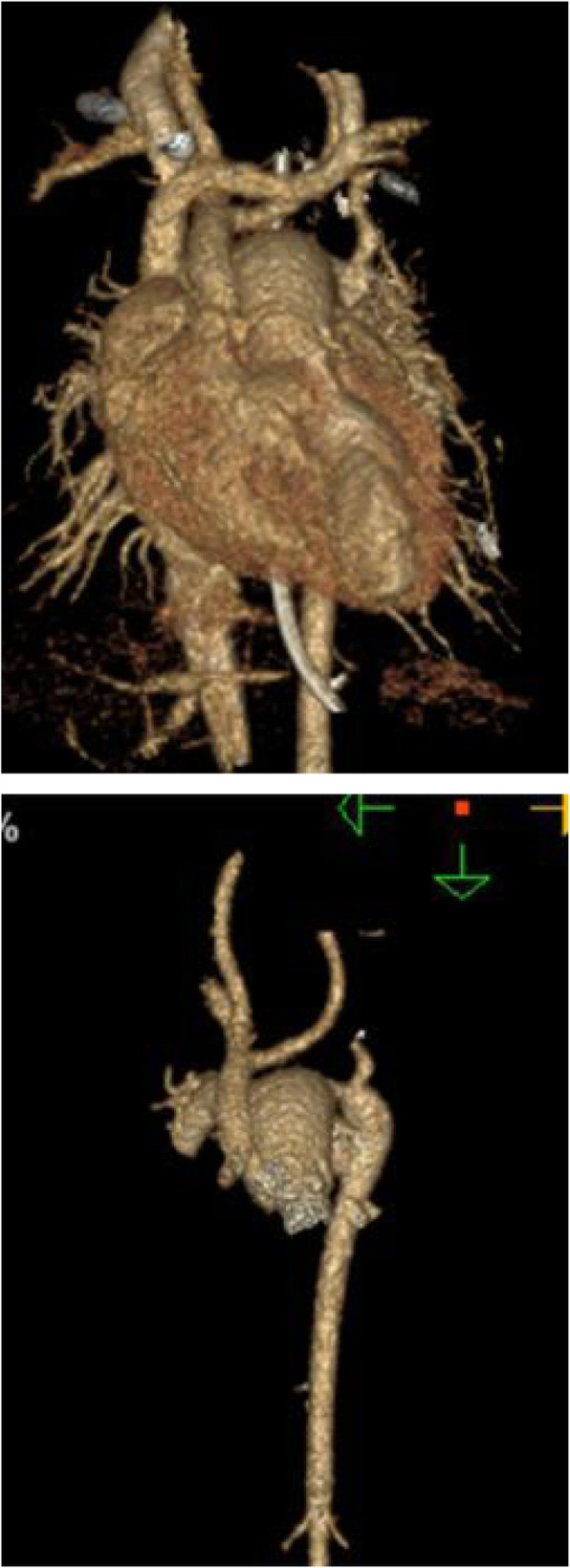
Preoperative three dimensional reconstruction images which showed significant mismatch of the great arteries

A 1-month old male infant weighing 3.6 kg was referred to our institution with symptoms of tachypnea and cyanosis. The patient’s mother refused to do the prenatal examination causing a delayed diagnosis. Echocardiography and computed tomography angiography (CTA) which are routinly peformed in our center for complex anatomical patterns before the operation revealed TGA with anterior-posterior-oriented great arteries, wide patent ductus arteriosus (PDA), type B IAA, ventricular septal defect (VSD) and pulmonary arterial hypertension (PHT), Fig. 1.

In October 2019, the patient underwent a single-stage primary surgical repair. The proximal descending aorta was separated from the distal aortic arch by approximately 2.6 cm. The left subclavian artery originated from the descending aorta which was perfused by a patent ductus arteriosus of 3.9 mm. Marked vessel discrepancy between the hypoplastic ascending aorta and the dilated main pulmonary artery (PA) due to the double size of PA than the ascending aorta was observed. No hypoplasia was noted in the right ventricle (RV) and the normal coronary pattern was observed.

A single-stage primary repair was performed through the midline sternotomy. Cardiopulmonary bypass (CPB) was introduced as both the ascending aorta and the PA which perfused the descending aorta through the wide patent ductus arteriosus were cannulated using a Y-shaped aortic inflow line. Cannulations of caval veins were then peformed with a right angle cannulation for the superior vena cava and a straight cannulation for the inferior vena cava. The aorta was cross-clamped at 30.2 °C core temperature during the systemic cooling and the myocardial protection was performed through infusions of the histidine-tryptophan-ketoglutarate (HTK) cardioplegic solution into the aortic root. The left heart venting was started by making a small incision on the atrial septum as there was no patent foramen ovale. As the RV was well developed, transpulmonary VSD closure was performed with a bovine pericardial patch after transecting the pulmonary trunk 5 mm above the annulus during the cooling process. At 20.5 °C the extracorporeal perfusion was stopped, and the arterial cannula was withdrawn from the pulmonary trunk. The aortic arch was then reconstructed under hypothermic circulatory arrest (HCA). The left subclavian artery was doubly ligated and transected during the reconstruction with the aim to relieve the tension of anastomosis. After complete excision of the ductal tissue, a longitudinal aortotomy was performed on the inner curvature of the ascending aorta. The end of the descending aorta was then directly anastomosed with the ascending aorta. After the completion of the aortic arch reconstruction systemic arterial perfusion was resumed.

The ascending aorta was then transected 5 mm above the coronary artery (CA) to perform an arterial switch operation of the transposed great arteries. As there were no variable coronary patterns both CA were excised with buttons of the aortic wall and implanted again to the proximal neoaorta. The miss-match between the two ends of neoaorta was then corrected during the anastomosis of neoaorta with an elliptical patch of bovine pericardium used for enlargement of the distal part of the ascending aorta. For next step, the incision of atrial septum was totally closed. Anastomosis of the neo-pulmonary trunk was at last accomplished using a glutaraldehyde-treated autologous pericardium leaving the neoaorta behind the pulmonary bifurcation, which adopted the Lecompte Manouvre. The patient was transferred to the intensive care unit with an open sternum and a peritoneal dialysis catheter placed in the abdominal cavity. The by-pass, cross-clamping and circulatory arrest times were 265, 184, and 33 min, respectively.

**Fig. 2:**
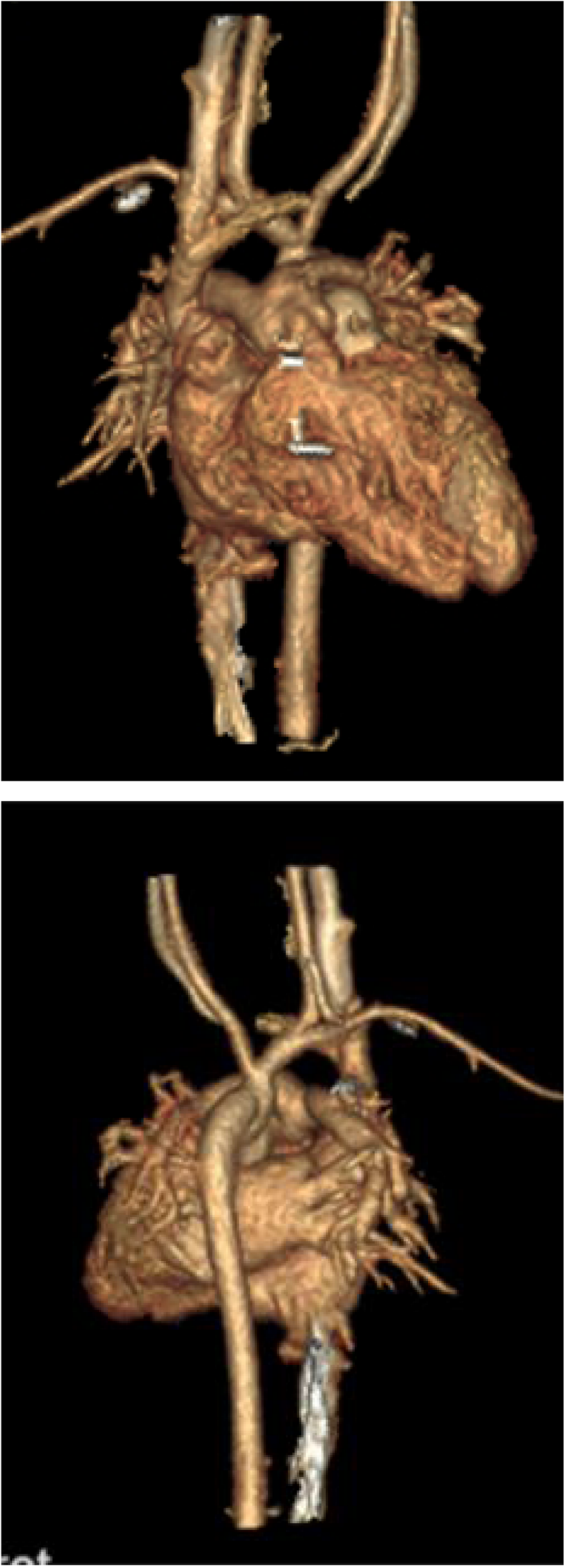
Postoperative three dimensional reconstruction images after the primary single-stage repair

The vasoactive-inotropic score (VIS), a predictor of morbidity and mortality in infants after cardiopulmonary bypass [3] was 17.5 (adrenaline 0.10μg/kg.min, noradrenaline 0.05μg/kg.min, isoprenaline 0.025μg/kg.min) at the first hour after the operation and 16 (adrenaline 0.10μg/kg.min, noradrenaline 0.03μg/kg.min, milrinone 0.3μg/kg.min) after 24 h. Nitroprusside was used to reduce afterload. N-terminal pro-brain natriuretic peptide (NT-proBNP) was 22,914.00 pg/ml the first day after the operation and decreased to 7101.00 pg/ml after 2 days. The ventilation time was 91 h and the patient stayed in the ICU for 12 days. Postoperative echocardiography and CTA showed excellent results as the systolic function of both ventricles was normal and there were no aortic or pulmonary regurgitations, Fig. 2. A maximum velocity of 1.7 m/s and 2.4 m/s was recorded at the neoaorta and neopulmonary anastomosis, respectively. The patient remained asymptomatic at 3 months after surgery confirmed with echocardiography demonstrating the normal systolic function of ventricles and trivial regurgitation for both aortic and pulmonary valves. The maximum velocity at the neoaorta and neopulmonary anastomosis was 1.8 m/s and 2.4 m/s, respectively.

## Discussion

TGA with aortic arch obstruction (AAO) is not a very common heart disease. The world experience with surgical repair of this complex congenital cardiac anomaly is limited to few studies, among these cases with combined TGA and IAA is even scarce. High mortality rates of the surgical repair for IAA in the combination of other conotruncal anomalies are also reported [1, 4, 5].

The two-staged repair including primary reconstruction of the aortic arch and excision of the ductal tissue with or without pulmonary artery banding through left thoracotomy followed by an arterial switch and intracardiac repair as well as pulmonary artery debanding after days through a midline sternotomy is one of the preferred surgical approaches to TGA with IAA and still is the choice of treatment for patients with RV hypoplasia or significant co-morbid conditions such as necrotizing enterocolitis or brain injury [6]. Konstantinov et al. [7] advocated in support of this approach to avoid prolonging the duration of CPB time necessary for the subsequent arterial switch operation and VSD closure. However, mortality rates for the two-staged repair are relatively high [8, 9]. The main cause of the high mortality rates is the presence of tubular hypoplasia of the aortic arch. The deleterious effects of pulmonary artery banding can lead to relatively high incidences of postoperative neoaortic regurgitation, systemic ventricular dysfunction, branch pulmonary artery distortion, and other factors [10–12]. But the requirement for a second-stage operation could arise earlier than anticipated if banding is not performed due to deteriorating congestive cardiac failure [10].

Nowadays due to a decrease in the operative mortality rates along with the development of neonatal cardiac surgery single-stage repair for TGA with IAA has been generally adopted as a preferred surgical plan [1, 4, 5, 10, 12–14]. Further this approach is also associated with several benefits such as the early re-establishment of the normal physiologic circulation and the avoidance of the complications of pulmonary artery banding.

However, the rate of RV hypoplasia for TGA with AAO has been reported to be higher than the isolated TGA [15–17]. The primary biventricular repair of TGA associated with AAO and RV hypoplasia has shown high mortality rates due to the postoperative right ventricular failure [2, 18, 19]. For neonatal patients associated with RV hypoplasia, a two-staged repair or a single-stage palliative repair including the aortic arch reconstruction, arterial switch operation (ASO) and intraluminal PA banding is likely to provide a better result than the primary single-stage repair by facilitating the development of the hypoplastic RV [20].

### Surgical management of primary one-stage repair

This process is truly a tough challenge to the surgeons as the lengthy surgery including the aortic arch reconstruction, ASO as well as the VSD closure can be far more complicated with complex coronary artery anatomies, mismatch of the great vessels and the presence of the subaortic obstruction. Hypoplasia of RV, severe pulmonary hypertension and complex coronary patterns associated with non-anteroposterior great arteries are considered as common additional risk factors for the surgery associated mortality [2].

#### VSD and ASD closure

VSD is often present in all patients. VSD and ASD are generally closed before arch reconstruction and ASO. The VSD closure is better performed either through the tricuspid valve or through the transected original pulmonary trunk, or both, with a bovine pericardial patch to save the autologous pericardium. Right ventriculotomy is generally avoided, especially for the patients with RV hypoplasia. Mohammadi et al. [11] considered transpulmonary VSD closure as a significant risk factor for neoaortic valve regurgitation. However, our patient who underwent a VSD closure through the original pulmonary valve is still doing well with trivial neoaortic regurgitation at a 3-month follow-up. The VSD patch is likely to affect the neoaortic valve if not trimmed to an appropriate pattern without excessive material [10]. In addition, the maligned conal septum is often excised for patients with double outlet right ventricle or subaortic obstruction as appropriate.

A fenestrated ASD patch is suggested for patients with a hypoplastic RV or severe pulmonary hypertension to smooth the course by allowing a restrictive interatrial communication. The temporary central aortopulmonary shunt has been reported as a useful rescue measure in patients who cannot wean successfully from CPB because of pulmonary hypertension [2, 17, 21]. In addition a one-way valved ASD patch has also been advocated in patients of RV hypoplasia or PHT [22].

#### Reconstruction of the aortic arch

After the performance of VSD closure, the aortic arch is reconstructed under HCA. The relatively long distance from the aortic arch to the descending aorta tends to increase the tension of anastomosis. Under some conditions, if required a transection of the left subclavian artery or a Gore-Tex tube graft could be helpful to circumvent this problem. Complete excision of the ductal tissue from the descending aorta is supported to relieve the tension of anastomosis and to prevent the recurrent coarctation from the retained ductal tissue [23].

For most patients, a direct end-to-end or end-to-side anastomosis between the aortic arch and the descending aorta is the appropriate choice. But for patients associated with various degrees of aortic arch hypoplasia, augmentation of the arch reconstruction should also be considered. A pulmonary homograft patch rather than a bovine pericardial patch which is potentially related to early fibrosis and arch re-obstruction is suggested for the enlargement of the aortic arch. Avoiding kinking of the patch by tailoring it in a short fashion at its concave portion is crucial in the case of the creation of infoldings at the inner curvature of the aortic arch [10].

#### Arterial switch operation

After the repair of IAA, arterial switch operation is performed under the resumed systemic arterial perfusion [19]. Except for the patients of side-by-side great arteries, the Lecompte procedure [24] is appropriate for most of the cases during which the proximal neoaorta is brought behind the pulmonary bifurcation. This maneuver can provide a smoother course for the neoaorta and it has been reported to reduce the probability of recurrent left or right ventricular outflow tract obstruction (LVOTO or RVOTO) [25]. Nevertheless, for patients of side-by-side great arteries, especially for those with complex coronary patterns, leaving the proximal neoaorta in front of the pulmonary bifurcation instead of the Lecompte maneuver must be considered to avoid the compression of coronary arteries by the dilated neopulmonary artery and its branches [16, 25, 26].

The pronounced mismatch between the two great vessels can add to the difficulties of reconstructing the neoaorta, which might lead to the distortion of the neoaortic valve, suture line tension, bleeding of the anastomosis, left bronchial compression, obstruction and possibly requires an artificial patch augmentation of the aortic arch [23]. Generally, two patches of the bovine pericardium or glutaraldehyde-treated autologous pericardium tailored cautiously to an appropriate shape to preserve the orientation of the transferred coronary buttons are used to widen the distal part of the ascending aorta and the proximal neopulmonary.

### Complications and re-interventions

As per the literature, postoperative complications, often blamed to the high re-intervention rate and mainly include recurrent coarctation, supravalvular aortic stenosis, neoaortic valve regurgitation, obstruction of the right heart system, coronary stenosis and other complications such as arrhythmia, bleeding, left phrenic nerve paralysis, left recurrent laryngeal nerve impairment, chylothorax, low cardiac output, renal failure, brain injury as well as sepsis. The chest X-ray, electrocardiogram, echocardiographic assessment, and diagnostic catheterizations can assist in decision making during post-operative care.

#### Recurrent coarctation and supravalvular aortic stenosis

Recoarctation has been reported in most of the relevant studies leading to a high re-intervention rate. In most cases, percutaneous balloon aortoplasty has shown efficiency for recurrent aortic coarctation making the open surgery a fallback solution. The end-to-side anastomosis performed by an enormous longitudinal aortotomy on the inner curvature of the ascending aorta can provide a generous lumen without obstruction. In addition, the use of an autologous patch instead of a bovine pericardial patch can also prevent the re-coarctating by avoiding the early fibrosis [10].

The postoperative supravalvular aortic stenosis has been reported as one of the reasons for operative mortality after the ASO might be related to the discrepancy in size between the dilated neoaortic root and the undersized ascending aorta. A wide patch augmentation of the entire arch is required in such cases [27].

#### Aortic valve regurgitation

Except for Mohammadi et al. [11], who considered transpulmonary VSD closure to be an important risk factor for the impaired valve function, most of the studies regarded aortic valve regurgitation as an infrequent postoperative complication requiring repeated surgery [2, 10, 12, 28]. The approach mentioned above to repair the subpulmonary VSD through the original pulmonary valve was adopted in our case and trivial regurgitation of the aortic valve was observed at a follow-up of 3 months.

The carefully trimmed VSD patch without excessive material impinged upon the neoaortic valve cusps and the homograft patches used for enlargement of the distal part of the ascending aorta to overcome the mismatch of the great arteries and the coronary arteries transferred to prevent the sinotubular junction from being interrupted are supposed to account for the low incidence of aortic valve regurgitation [12]. In contrast, Christoph et al. [10] considered an excessively proximal transection of the original PA and the additional material used during the reconstruction of the neoaorta was more likely to result in various degrees of distortion of the sino-tubular junction which might interfere with the valve integrity.

#### Obstruction of the right heart system

Right-sided obstruction consisting of RVOTO, stenosis of the pulmonary valve, pulmonary trunk and its branches is one of the main indications for the re-intervention [28]. Postoperative pulmonary valve stenosis and RVOTO are common in patients with the combined TGA and AAO, who usually have small aortic annuli and narrow right ventricular outflow tracts [2, 27, 29]. In addition, an aberrant right subclavian obstruction artery is considered as one of the risk factors for RVOTO [1]. Some studies have also demonstrated that no significant risk factors of postoperative RVOTO were found in patients with Taussig-bing anomalies and AAO, considering the combination with various coronary anomalies, non-anteroposterior great arteries, and the preoperative organic subaortic stenosis [12, 30].

Most patients with postoperative right-sided obstruction benefit from the percutaneous balloon valvuloplasty and angioplasty. Transannular patch enlargement of the RVOT and patch angioplasty of the pulmonary trunk or branches are generally considered as a fallback choice when the percutaneous intervention is unsuccessful [10, 12, 28]. A double-barrel technique with a valved xenograft conduit from the RV infundibulum to the pulmonary trunk can be used in presence of aberrant coronary arteries crossing the ventriculo-arterial junction which may preclude the patch enlargement [31, 32]. For the purpose of preventing the thickening and stiffening of the fibrous tissues from adding to the difficulties of postoperative percutaneous interventions, our center’s experience is to perform the percutaneous interventions, which is generally available 1 month after the primary operation by lowering the threshold of a pressure gradient to 30 mmHg. Although it seems to lead to a relatively high re-intervention rate, we speculate that the re-operation rate for right-sided obstruction might decrease consequently.

An excision of hypertrophic conal septum or division of subvalvular trabeculations is occasionally required to open up the RVOT, which can be performed through the original aortic valve or a longitudinal incision at the RVOT [2, 10]. With the ability of differentiation into arterial vascular wall, fresh autologous pericardium can be used to reconstruct the defects of the neopulmonary artery after excising both coronary arteries with buttons, which might reduce the possibility of recurrent RVOTO [33]. Additionally, for patients undergone a two-staged repair, the RVOTO might further develop after the primary palliative surgery during the PA banding and aortic arch reconstruction were performed [8].

#### Coronary stenosis

Obstruction of the relocated coronaries which might be compressed by the dilated neopulmonary arteries or be distorted because of technical problems during the ASO is one of the primary indications for re-operation. Especially for patients with side-by-side great arteries, the performance of Lecompte maneuver might lead to coronary compression by the anteriorly translocated pulmonary arteries. Stenosed coronary arteries can be enlarged with fresh autologous patches consisting of unimportant superficial veins such as a portion of a saphenous or innominate vein [34].

## Conclusions

In summary, a single-stage repair with VSD closure, reconstruction of aortic arch and ASO might be an applicable approach for most of the patients with combined TGA and IAA. The high re-intervention rate for recurrent coarctation, supravalvular aortic stenosis, neoaortic valve regurgitation, obstruction of the right heart system and coronary stenosis has been reported. Long term follow-up is required and early percutaneous interventions might reduce the repeated surgery rates for right-sided obstruction.

## Data Availability

Data sharing is not applicable to this article as no datasets were generated or analysed during the current study.

## Abbreviations

AAO: Aortic arch obstruction
ASO: Arterial switch operation
CA: Coronary artery
CPB: Cardiopulmonary bypass
CTA: Computed tomography angiography
HCA: Hypothermic circulatory arrest
HTK: Histidine-tryptophan-ketoglutarate
IAA: Interruption of the aortic arch
LVOTO: Left ventricular outflow tract obstruction
NT-proBNP: N-terminal pro-brain natriuretic peptide
PA: Pulmonary artery
PDA: Patent ductus arteriosus
PHT: Pulmonary arterial hypertension
RV: Right ventricle
RVOTO: Right ventricular outflow tract obstruction
TGA: Transposition of the great arteries
VSD: Ventricular septal defect
VIS: Vasoactive-inotropic score
